# Association of systolic blood pressure variability with remote ischemic conditioning in acute ischemic stroke

**DOI:** 10.1038/s41598-024-66572-2

**Published:** 2024-07-06

**Authors:** Yu Cui, Yue-Xin Ning, Ji-Ru Cai, Nan-Nan Zhang, Hui-Sheng Chen

**Affiliations:** 1 Department of Neurology, General Hospital of Northern Theater Command, Shenyang, 110016 China; 2 Department of Life Science and Biopharmaceutics, Shenyang Pharmaceutical University, Shenyang, China; 3 Department of Neurology, Postgraduate Training Base of Jinzhou Medical University in the General Hospital of Northern Theater Command, Shenyang, China

**Keywords:** Acute ischemic stroke, Remote ischemic conditioning, Systolic blood pressure variability, Functional outcome, Medical research, Neurology

## Abstract

Systolic blood pressure variability (SBPV) is associated with outcome in acute ischemic stroke. Remote ischemic conditioning (RIC) has been demonstrated to be effective in stroke and may affect blood pressure. Relationship between SBPV and RIC treatment after stroke warrants investigation. A total of 1707 patients from per-protocol analysis set of RICAMIS study were included. The SBPV was calculated based on blood pressure measured at admission, Day 7, and Day 12. (I) To investigate the effect of SBPV on efficacy of RIC in stroke, patients were divided into High and Low categories in each SBPV parameter. Primary outcome was excellent functional outcome at 90 days. Compared with Control, efficacy of RIC in each category and interaction between categories were investigated. (II) To investigate the effect of RIC treatment on SBPV, SBPV parameters were compared between RIC and Control groups. Compared with Control, a higher likelihood of primary outcome in RIC was found in high category (max–min: adjusted risk difference [RD] = 7.2, 95% CI 1.2–13.1, *P* = 0.02; standard deviation: adjusted RD = 11.5, 95% CI 1.6–21.4, *P* = 0.02; coefficient of variation: adjusted RD = 11.2, 95% CI 1.4–21.0, *P* = 0.03). Significant interaction of RIC on outcomes were found between High and Low standard deviations (adjusted *P* < 0.05). No significant difference in SBPV parameters were found between treatment groups. This is the first report that Chinese patients with acute moderate ischemic stroke and presenting with higher SBPV, who were non-cardioemoblic stroke and not candidates for intravenous thrombolysis or endovascular therapy, would benefit more from RIC with respect to functional outcomes at 90 days, but 2-week RIC treatment has no effect on SBPV during hospital.

## Introduction

Reperfusion therapies, such as intravenous thrombolysis and endovascular thrombectomy have been standard treatments for acute ischemic stroke^1,2^, however, the strategy is limited by strict eligibility criteria and imperfect treatment effect^3,4^. It has been a hot topic to explore cerebral protection as an adjunct therapy to improve prognosis of stroke over the past years^5^. Up to date, few cerebral protective strategies have been translated from preclinical or clinical trials to clinical practice^6^.

Remote ischemic conditioning (RIC), intermittently blocking the blood flow of limbs and producing transient ischemic with the intention of protecting brain, has been demonstrated to improve neurological function after stroke in both animal model and clinical trial^7,8^. The Remote Ischemic Conditioning for Acute Moderate Ischemic Stroke (RICAMIS) trial demonstrated that RIC treatment initiated within 48 h of stroke onset safely and significantly improved excellent functional outcome at 90 days among patients with acute moderate ischemic stroke who did not receive any reperfusion therapy^9^. Systolic blood pressure variability (SBPV) was previously reported to be strongly associated with functional outcome after acute ischemic stroke^10,11^. An exploratory study demonstrated that 4-week RIC treatment may reduce blood pressure in partial hypertension patients^12^. Given the potential relationship among blood pressure, functional outcome after stroke, and RIC treatment, it is worth exploring whether there is an association between SBPV and effect of RIC treatment on functional outcomes in acute ischemic stroke, and whether 2-week RIC treatment will result in change of SBPV, which further affect RIC efficacy.

In this context, we hypothesized that SBPV may be associated with the effect of RIC treatment on functional outcomes in patients with acute ischemic stroke.

## Materials and methods

### Study design and participants

The current study was a post hoc analysis of the RICAMIS study and designed according to the CONSORT guideline and in accordance with the ethical principles that have their origins in the Declaration of Helsinki and its subsequent amendments. Details on the design and protocol of RICAMIS have been published^9,13^. In brief, RICAMIS trial was a multicenter, open-label, blinded-end point, randomized clinical trial enrolling 1893 patients between December 26, 2018, and April 19, 2021, to assess the efficacy of RIC in patients with acute moderate ischemic stroke. Eligible patients were 18 years and older, had been functioning independently before stroke (modified Rankin Scale [mRS] scores, 0–1; range, 0 [no symptoms] to 6 [death]) and diagnosed with acute moderate ischemic stroke (National Institute of Health Stroke Scale [NIHSS] scores at admission, 6–16) within 48 h after stroke onset. Exclusion criteria were patients who received intravenous thrombolysis or endovascular therapy, had any contraindication for RIC treatment, or had cardiogenic embolism. All study procedures were reviewed and approved by Ethics Committee of General Hospital of Northern Theater Command (approval number: k 2018[43]), and written informed consents were obtained from patients or their legally authorized representatives. The study was registered with ClinicalTrials.gov (NCT03740971). Considering the effect of RIC treatment as protocol (defined as 80–120% completion of a 10–14 days) on blood pressure was investigated, patients in per-protocol analysis set of RICAMIS were included in the post hoc analysis.

### Procedures

The post hoc analysis included two parts. (I) We explored the association between SBPV parameter (max–min, standard deviation, and coefficient of variation) and effect of RIC treatment on functional outcomes^14^. The SBPV was visit-to-visit calculated by systolic blood pressure at admission, 7 days, and 12 days after randomization, which were measured by nurse at admission or eight o’clock (7 days and 12 days) and recorded in the electronic data capture system. According to the cutoff value of each SBPV parameter determined by receiver operating characteristic curve analysis, eligible patients were divided into High and Low categories in each SBPV parameter. In each category, patients were subdivided into RIC and Control groups according to whether they received RIC treatment as an adjunct to usual care based on current guideline^3^. RIC treatment was performed by 5 cycles of cuff inflation (200 mm Hg for 5 min) and deflation (for 5 min), for a total procedure time of 50 min, twice daily for 10–14 days. Further detail of RIC treatment has been described in a previous report^9^. (II) We explored the effect of RIC treatment on each SBPV parameter, compared with usual care based on current guideline^3^.

### Outcomes

(I) The primary outcome was excellent functional outcome at 90 days, defined as a mRS score of 0–1. The secondary outcomes were favorable functional outcome at 90 days, defined as a mRS score of 0–2; a shift in measure of functional outcome according to distribution on the ordinal mRS score at 90 days. The mRS score at 90 days was assessed in person or by telephone by trained and certified assessors in each center who were unaware of treatment allocation or clinical details. (II) The measurements of SBPV parameters were the outcomes in this part.

### Statistical analysis

The post hoc analysis was performed in the per-protocol analysis set of RICAMIS. The similar characteristics of populations in per-protocol analysis set and full analysis set addressed selection bias. Furthermore, given potential imbalance between treatment groups after classification by cutoff values of SBPV, primary analyses in the study were adjusted analyses.

Baseline characteristics of patients and results of outcome comparison were described by different methods. For the baseline characteristics of eligible patients, we summarized continuous variables as medians (interquartile range [IQR]) and categorical variables as frequencies (percentages). For the treatment effect of outcomes, such as excellent functional outcome and favorable functional outcome at 90 days, we estimated the absolute number of events, and absolute difference (risk difference, RD). For the treatment effect of mRS score distribution at 90 days, we estimated the odds ratio (OR). The two-sided 95% confidence intervals (CI) and *P* values were also generated with treatment effect metrics.

(I) First, probability of primary outcome was respectively calculated in RIC and Control groups through binary logistic regression analysis, and best-fit lines with their 95% CIs were respectively drawn according to probability and each SBPV parameter according to treatment group. Second, we respectively detected the association between treatments and functional outcomes in High and Low categories of each SBPV parameter. Generalized linear models, which had a binomial distribution, were performed to generate the RD. Ordinal regression analysis was performed to generate the OR. Third, the interactions of treatment effects on outcomes were assessed between two categories. The assessments of SBPV and effect of treatments on functional outcomes were conducted by generalized linear model or ordinal regression analysis with treatment groups, categories, and their interaction terms as independent variables, and the *Pint* values were presented for interaction terms. (II) We compared SBPV parameters between RIC and Control groups. General linear model was performed to generate the beta coefficient.

Adjusted analyses included baseline variables, which showed difference between treatment groups or categories with *P* values < 0.1. Sensitivity analysis for efficacy of RIC in each category was performed to address the imbalanced baseline characteristics and sample size between treatment groups by propensity score matching. Details of adjusted and sensitivity analyses were shown in Expanded Methods of Supplementary Material.

All analyses presented were exploratory, and all *P* values were nominal. No multiple comparisons were performed. Two-sided *P* values < 0.05 were considered significant. All statistical analyses were performed using the SPSS software (version 26.0, IBM) and R software (version 4.1.0).

## Results

A total of 1707 patients from per-protocol analysis set of RICAMIS trial were included in this post hoc analysis, including 808 in the RIC group and 899 in the Control group. Patients in per-protocol and full analysis sets were similar (Table S1). Baseline characteristics of patients in per-protocol were balanced between treatment groups, except for presumed stroke cause (Table S2). The cutoff values for SBPV parameters were 15.5 mmHg for max–min (990 in the high category and 717 in the low category), 16.89 for standard deviation (402 in the high category and 1305 in the low category), and 11.52 for coefficient of variation (401 in the high category and 1306 in the low category), respectively. Baseline characteristics of patients among treatment groups in two categories were shown in Tables S3–S5.

(I) The association between SBPV parameters and efficacy of RIC in stroke

**Figure 1:**
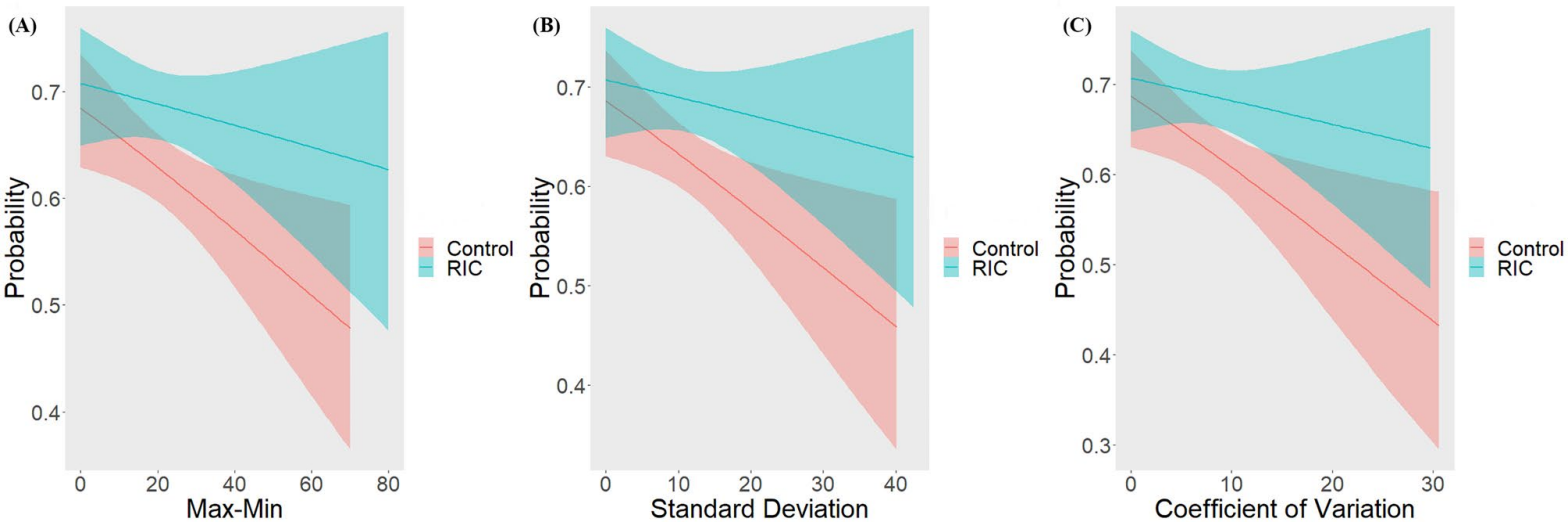
Probability of Primary Outcome According to SBPV Parameters. SBPV, systolic blood pressure variability; RIC, remote ischemic conditioning. Patients in Control group received guideline-based usual care alone without RIC treatment. Primary outcome was excellent functional outcome, which was defined as modified Rankin Scale score of 0–1 at 90 days.

First, probability of mRS score 0–1 at 90 days decreased as SBPV increased in both RIC and Control groups. Compared with Control group, the probability was higher in the RIC group and the gap of probability between treatment groups became large as SBPV increased more (Fig. 1).

**Figure 2:**
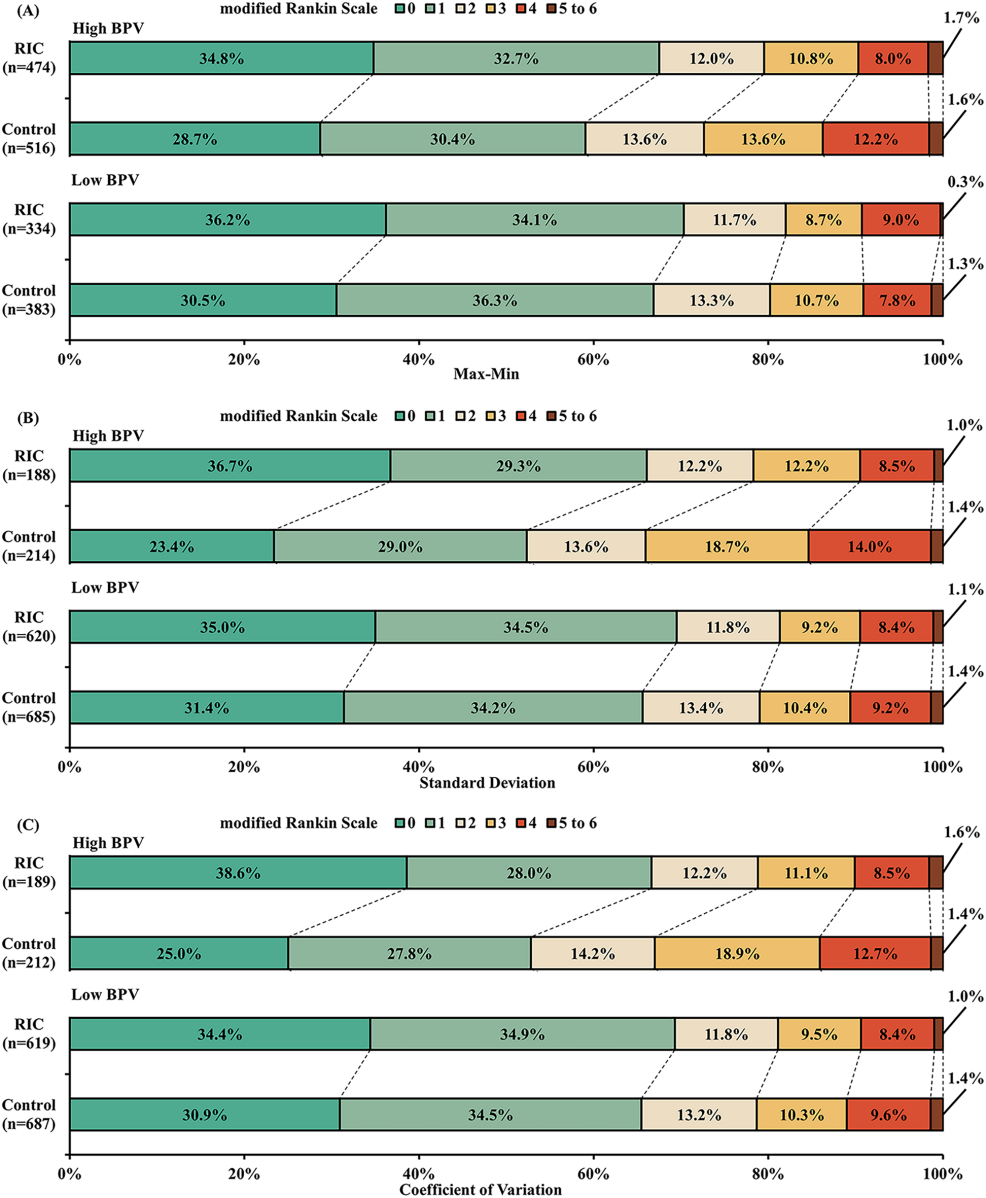
Distribution of modified Rankin Scale Score at 90 Days According to Different Categories in Each SBPV Parameter. The percentages of patients are shown according to the raw distribution of mRS scores at 90 days. Scores on the mRS range from 0 to 6. 0 = no symptoms, 1 = symptoms without clinically significant disability, 2 = slight disability, 3 = moderate disability, 4 = moderately severe disability, 5 = severe disability, and 6 = death. The Control group included patients who received guideline-based usual care alone without RIC treatment. RIC, remote ischemic conditioning; SBPV, systolic blood pressure variability.

Second, functional outcomes between RIC and Control in each category were compared (Table 1 and Fig. 2). Compared with Control group, RIC increased likelihood of mRS score 0–1 at 90 days in patients with High max–min (adjusted RD, 7.2%; 95% CI 1.2–13.1%; *P* = 0.02), High standard deviation (adjusted RD, 11.5%; 95% CI 1.6–21.4%; *P* = 0.02), and High coefficient of variation (adjusted RD, 11.2%; 95% CI 1.4–21.0%; *P* = 0.03). Similar results were found in the secondary outcomes. Notably, we found significant interaction effects of intervention (RIC or Control) by High vs Low standard deviation for functional outcomes including mRS 0–1 (*P*_int_ = 0.04), mRS 0–2 (*P*_int_ = 0.02), and mRS distribution at 90 days (*P*_int_ = 0.02). A similar interaction for the efficacy of RIC on secondary outcomes were also found between High vs Low coefficient of variation.

**Table 1:** Comparison of Functional Outcomes between Treatment Groups According to Category in Each SBPV Parameter.

SBPV parameter	Outcomes	Category	Groups	Number of events (%)	Treatment effect metric	Unadjusted			Adjusted		
						Treatment difference (95% CI)	*P* Value	*P*_*int*_ Value	Treatment difference (95% CI)	*P* Value^a^	*P*_*int*_ Value^b^
Max–min	mRS score 0 to 1 at 90 day^c^	High (N = 990)	RIC (N = 474)	320 (67.5)	RD,%^d^	8.4 (2.4 to 14.4)	0.006	0.29	7.2 (1.2 to 13.1)	0.02	0.27
			Control (N = 516)	305 (59.1)							
		Low (N = 717)	RIC (N = 334)	235 (70.4)		3.5 (− 3.3 to 10.3)	0.31		3.9 (− 3.0 to 10.7)	0.27	
			Control (N = 383)	256 (66.8)							
	mRS score 0 to 2 at 90 day^c^	High (N = 990)	RIC (N = 474)	378 (79.7)	RD,%^d^	7.1 (1.8 to 12.4)	0.009	0.19	6.7 (1.5 to 12.0)	0.01	0.19
			Control (N = 516)	375 (72.7)							
		Low (N = 717)	RIC (N = 334)	274 (82.0)		1.9 (− 3.9 to 7.6)	0.52		2.4 (− 3.4 to 8.1)	0.42	
			Control (N = 383)	307 (80.2)							
	mRS score at 90 d ^c^	High (N = 990)	RIC (N = 474)	N/A	OR^e^	1.39 (1.11 to 1.74)	0.004	0.40	1.34 (1.07 to 1.68)	0.01	0.39
			Control (N = 516)	N/A							
		Low (N = 717)	RIC (N = 334)	N/A		1.22 (0.94 to 1.59)	0.14		1.23 (0.95 to 1.61)	0.12	
			Control (N = 383)	N/A							
Standard deviation	mRS score 0 to 1 at 90 day^c^	High (N = 402)	RIC (N = 188)	124 (66.0)	RD,%^d^	13.6 (4.1 to 23.1)	0.005	0.08	11.5 (1.6 to 21.4)	0.02	0.04
			Control (N = 214)	112 (52.3)							
		Low (N = 1305)	RIC (N = 620)	431 (69.5)		4.0 (− 1.1 to 9.0)	0.13		4.0 (− 1.1 to 9.0)^f^	0.13 f.	
			Control (N = 685)	449 (65.5)							
	mRS score 0 to 2 at 90 day^c^	High (N = 402)	RIC (N = 188)	147 (78.2)	RD,%^d^	12.3 (3.6 to 21.0)	0.005	0.05	10.6 (1.8 to 19.3)	0.02	0.02
			Control (N = 214)	141 (65.9)							
		Low (N = 1305)	RIC (N = 620)	505 (81.5)		2.5 (− 1.8 to 6.8)	0.26		2.5 (− 1.8 to 6.8)^f^	0.26 f.	
			Control (N = 685)	541 (79.0)							
	mRS score at 90 day^c^	High (N = 402)	RIC (N = 188)	N/A	OR^e^	1.83 (1.28 to 2.61)	0.001	0.02	1.62 (1.13 to 2.34)	0.009	0.02
			Control (N = 214)	N/A							
		Low (N = 1305)	RIC (N = 620)	N/A		1.18 (0.97 to 1.44)	0.10		1.18 (0.97 to 1.44)^f^	0.10 f.	
			Control (N = 685)	N/A							
Coefficient of variation	mRS score 0 to 1 at 90 day^c^	High (N = 401)	RIC (N = 189)	126 (66.7)	RD,%^d^	13.8 (4.3 to 23.3)	0.004	0.07	11.2 (1.4 to 21.0)	0.03	0.06
			Control (N = 212)	112 (52.8)							
		Low (N = 1306)	RIC (N = 619)	429 (69.3)		3.9 (− 1.1 to 9.0)	0.13		3.9 (− 1.1 to 9.0)^f^	0.13 f.	
			Control (N = 687)	449 (65.4)							
	mRS score 0 to 2 at 90 day^c^	High (N = 401)	RIC (N = 189)	149 (78.8)	RD,%^d^	11.9 (3.3 to 20.5)	0.007	0.06	8.6 (− 0.3 to 17.5)	0.06	0.04
			Control (N = 212)	142 (67.0)							
		Low (N = 1306)	RIC (N = 619)	503 (81.3)		2.7 (− 1.7 to 7.0)	0.23		2.7 (− 1.7 to 7.0)^f^	0.23 f.	
			Control (N = 687)	540 (78.6)							
	mRS score at 90 day^c^	High (N = 401)	RIC (N = 189)	N/A	OR^e^	1.80 (1.26 to 2.57)	0.001	0.03	1.58 (1.09 to 2.27)	0.01	0.03
			Control (N = 212)	N/A							
		Low (N = 1306)	RIC (N = 619)	N/A		1.18 (0.97 to 1.44)	0.09		1.18 (0.97 to 1.44)^f^	0.09 f.	
			Control (N = 687)	N/A							

*CI* confidence interval, *mRS* modified Rankin Scale, *N/A* not applicable, *OR* odds ratio, *RD* risk difference, *RIC* remote ischemic conditioning, *SBPV* systolic blood pressure variability.

^a^Adjusted for covariates compared between RIC and Control group with *P* value < 0.1 in the SBPV category.

^b^Adjusted for covariates compared between High and Low SBPV categories with *P* value < 0.1.

^c^mRS scores range from 0 to 6: 0 = no symptoms, 1 = symptoms without clinically significant disability, 2 = slight disability, 3 = moderate disability, 4 = moderately severe disability, 5 = severe disability, and 6 = death.

^d^Calculated using generalized linear model.

^e^Calculated using ordinal regression analysis.

^f^Without adjustment due to no covariates compared between RIC and Control group with *P* value < 0.1 in the category.

Third, in the sensitivity analysis for primary outcome, baseline characteristics between matched treatment groups were balanced (Tables S6–S8). The comparisons of mRS score 0–1 at 90 days between matched treatment groups in each category were consistent with those in the primary analysis (Table 2).

**Table 2:** Sensitivity for Primary Outcome Comparison between Treatments Groups in Each Category after Propensity Score Matching.

SBPV parameter	Category	No.(%) of events		Treatment difference (95% CI)^a^	*P* value	*P*_int_ value^b^
		RIC group	Control group			
Max–Min	High (n = 828)	279/414 (67.4)	248/414 (59.9)	7.5 (1.0 to 14.0)	0.03	0.63
	Low (n = 646)	228/323 (70.6)	219/323 (67.8)	2.8 (− 4.3 to 9.9)	0.44	
Standard Deviation	High (n = 310)	104/155 (67.1)	85/155 (54.8)	12.3 (1.5 to 23.0)	0.03	0.29
	Low (n = 1048)	408/584 (69.9)	378/584 (64.7)	5.1 (− 0.2 to 10.5)	0.06	
Coefficient of Variation	High (n = 304)	104/152 (68.4)	85/152 (55.9)	12.5 (1.7 to 23.3)	0.03	0.17
	Low (n = 1140)	400/570 (70.2)	378/570 (66.3)	3.9 (− 1.5 to 9.3)	0.16	

*CI* confidence interval, *RIC* remote ischemic conditioning, *SBPV* systolic blood pressure variability.

^a^Calculated using generalized linear model and presented by risk difference.

^b^*P*_int_ value means the *P* value for interaction.

(II) The effect of RIC treatment on SBPV

**Figure 3:**
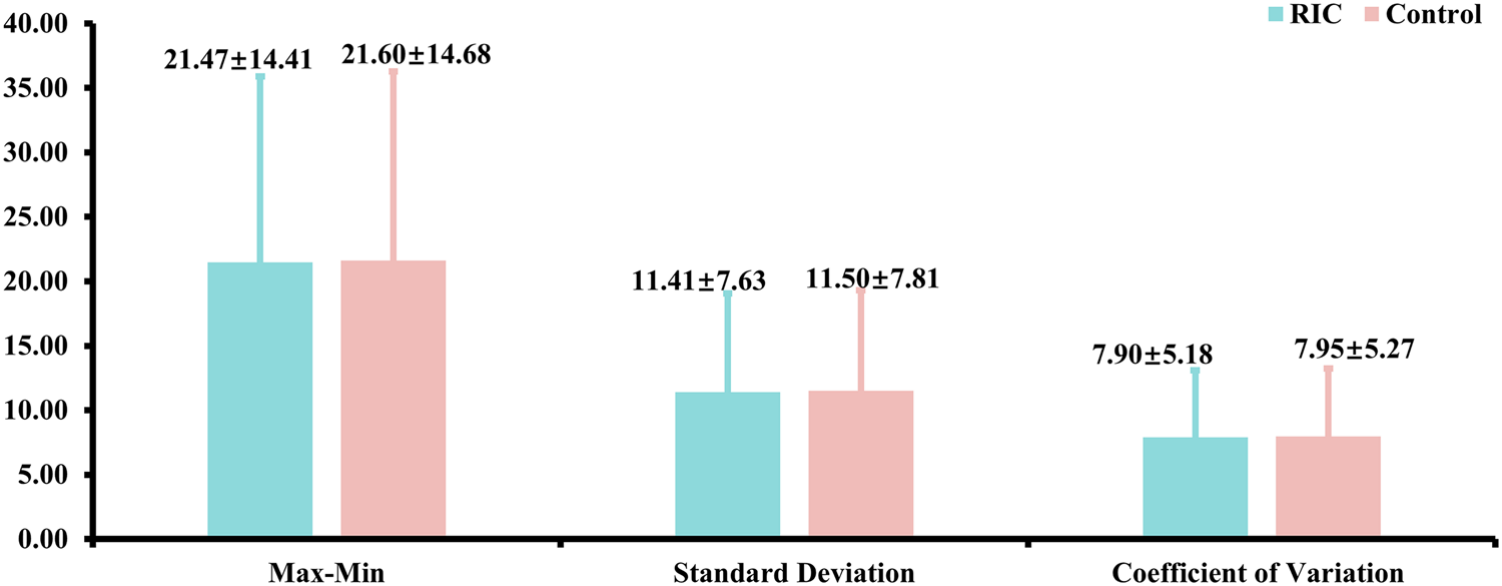
Comparison of Change in SBPV Parameters between Treatment Groups. The Control group included patients who received guideline-based usual care alone without RIC treatment. RIC, remote ischemic conditioning; SBPV, systolic blood pressure variability. There were no significant difference between RIC and Control groups in SBPV parameters (max–min: beta coefficient = − 0.004, 95% CI − 0.048 to 0.040, *P* value = 0.86; standard deviation: beta coefficient = − 0.006, 95% CI − 0.056 to 0.043,* P* value = 0.81; Coefficient of variation: beta coefficient = − 0.005, 95% CI − 0.050 to 0.040,* P* value = 0.83).

Three SBPV parameters were compared between RIC and Control groups. No significant difference was found in max–min, standard deviation, coefficient of variation of systolic blood pressure during hospital between groups (Fig. 3).

## Discussion

In this post hoc analysis of RICAMIS trial, we found that SBPV was significantly associated with the efficacy of RIC treatment after acute ischemic stroke. Compared with Control group, the likelihood of excellent functional outcomes at 90 days in RIC group was significant higher in patients with higher SBPV parameters. Additionally, 2-week RIC treatment did not significantly affect SBPV.

This is the first study to explore the relationship between SBPV and RIC treatment in patients with acute moderate ischemic stroke. Given the predictive ability of systolic BPV in stroke outcome rather than diastolic BPV or mean BPV, we explored the association between SBPV and RIC efficacy in acute ischemic stroke^15^. In patients with higher SBPV, we found that RIC significantly improved the functional outcomes compared with usual care alone, suggesting the significant effect of SBPV on RIC efficacy. Previous studies demonstrated that high SBPV was associated with poor functional outcome after acute ischemic stroke^11,16^. After acute moderate ischemic stroke, the cerebral autoregulation was impaired^17^. Increased blood pressure variability could produce deleterious fluctuations in cerebral perfusion which maintained the cerebral blood flow^18^. RIC treatment was proven to be an effective way to improve cerebral perfusion in patients with ischemic stroke^19^. In this context, we infer that RIC treatment may more significantly improve cerebral perfusion regulation in patients with higher SBPV, resulting in patients benefiting more from RIC treatment.

Given that RIC treatment were previously reported to affect blood pressure, we tried to investigate the effect of RIC treatment on SBPV. In the current study, we found 2-week RIC treatment could not significantly affect SBPV after acute ischemic stroke compared with usual care alone. The results were inconsistent with those in the previous study, which showed RIC treatment could significantly reduce blood pressure^12^. The neutral results may be attributed to different target population and time course of RIC treatment in two studies. First, a strict inclusion criteria was conducted to screen participants according to the history of hypertension and use of antihypertensive medications in the previous study. Second, 4-week RIC treatment in the previous study was longer than that in RICAMIS study (2 week). Third, the occurrence of acute ischemic stroke may influence the effect of RIC treatment in blood pressure, given that acute hypertensive response occurred in more than 50% of patients and blood pressure spontaneously decreased over several days^20,21^. Fourth, blood pressure variability couldn’t absolutely represent the change in blood pressure. Collectively, the effect of RIC treatment on blood pressure variability in acute ischemic stroke warrants further investigation.

In the current study, we found that higher SBPV was associated with more benefit from RIC treatment, while 2-week RIC treatment had no effect SBPV after acute ischemic stroke. SBPV was previously reported to be associated with poor outcome after acute ischemic stroke^11,16^. Considering the neutral effect of RIC treatment on SBPV and more benefit from RIC treatment in patients with higher SBPV, we inferred the efficacy of RIC treatment in stroke distinguished by SBPV may be attributed to the influence of SBPV on cerebral perfusion rather than the influence of RIC on SBPV. However, in the current study, the SBPV was calculated only based on three times’ measurement, but not more extensive set of measurements, for example, measurements for 7 continuous days in the previous study^11^. The limited measurements may not more accurately reflect actual SBPV, which will affect the current findings. Thus, the association between SBPV and RIC efficacy needs further investigation by more extensive blood pressure measurement in the future.

There were several limitations in the current study. First, in this post hoc analysis, an imbalanced sample size between treatment groups may have rendered our study underpowered. Although we performed sensitivity analysis to reduce the unbalanced bias by propensity score matching, the statistical power was also hampered by smaller sample size in treatment groups and categories. Second, patients with baseline systolic blood pressure more than 180 mmHg were excluded from RICAMIS trial, and hence, no data were available for those with severe hypertension at admission. Similarly, as RICAMIS trial excluded patients who were diagnosed as cardioembolic stroke or had received intravenous thrombolysis or endovascular therapy, the findings couldn’t represent the association of SBPV and RIC treatment efficacy in patients with cardioembolic stroke or receiving reperfusion treatments. Third, given that SBPV was a continuous parameter, it may limitedly influence the association between SBPV and outcomes through analyzing it as dichotomous variables. Fourth, it was worth exploring the association between efficacy of RIC treatment and the SBPV calculated with more time point or in the acute phase. Fifth, relatively less proportion of patients with sufficient 14-day RIC treatment and different characteristics of patients from previous study may result in the neutral effect of RIC treatment on SBPV. Sixth, the potential effect of antihypertensive drugs on SBPV should not be ignored, which may bias the association between SBPV and cerebral perfusion^22^. Finally, the generalizability of the results would need to be validated in other cohorts, particularly in a non-Chinese population. We interpret our findings with caution due to the exploratory nature of this post hoc analysis.

In conclusion, this post hoc exploratory analysis of RICAMIS trial suggests that Chinese patients with acute moderate ischemic stroke and presenting with high in-hospital SBPV, who were non-cardioembolic and not candidates for intravenous thrombolysis or endovascular therapy, would benefit more from RIC treatment with respect to functional outcomes at 90 days, but 2-week RIC treatment could not significant affect SBPV. These findings warrant further confirmation.

### Supplementary Information

Supplementary Information.

## Data Availability

The data that support findings of study are available from the corresponding author on reasonable request.
