# Supplementary material for: Association of systolic blood pressure variability with remote ischemic conditioning in acute ischemic stroke
Source: Sci Rep. 2024 Jul 6;14:15562. doi: 10.1038/s41598-024-66572-2 (PMC11227509; doi:10.1038/s41598-024-66572-2)
Supplement: Supplementary file 1 — Supplementary Information. [file 41598_2024_66572_MOESM1_ESM.docx]

**Supplementary Materials**

**“Association of systolic blood pressure variability with remote ischemic conditioning in acute ischemic stroke”**

**Yu Cui, Ph.D.; Yue-Xin Ning, M.M.; Ji-Ru Cai, M.M.; Nan-Nan Zhang, M.M.; Hui-Sheng Chen, M.D., Ph.D.**

**content**

[**CONSORT Checklist** 2](#_Toc146790187)

[**Expanded Methods** 4](#_Toc146790188)

[**Table S1 Baseline Characteristics between Different Analysis Sets in RICAMIS.** 5](#_Toc146790189)

[**Table S2 Baseline Characteristics between Groups in the Current Analysis.** 6](#_Toc146790190)

[**Table S3 Baseline Characteristics between Treatment Groups in Different Max-Min Categories.** 8](#_Toc146790191)

[**Table S4 Baseline Characteristics between Treatment Groups in Different Standard Deviation Categories.** 10](#_Toc146790192)

[**Table S5 Baseline Characteristics between Treatment Groups in Different Coefficient of Variation Categories.** 12](#_Toc146790193)

[**Table S6 Baseline Characteristics between Treatment Groups in Different Max-Min Categories after PSM.** 14](#_Toc146790194)

[**Table S7 Baseline Characteristics between Treatment Groups in Different Standard Deviation Categories after PSM.** 16](#_Toc146790195)

[**Table S8 Baseline Characteristics between Treatment Groups in Different Coefficient of Variation Categories after PSM.** 18](#_Toc146790196)

# **CONSORT Statement—CONSORT 2010 checklist of information to include when reporting a randomised trial**

| **Section/Topic** | **Item No** | **Checklist item** | **Reported on page No** |
| --- | --- | --- | --- |
| **Title and abstract** | | | |
|  | 1a | Identification as a randomised trial in the title | Not applicable |
|  | 1b | Structured summary of trial design, methods, results, and conclusions (for specific guidance see CONSORT for abstracts) | 2 |
| **Introduction** | | | |
| Background and objectives | 2a | Scientific background and explanation of rationale | 3 |
|  | 2b | Specific objectives or hypotheses | 4 |
| **Methods** | | | |
| Trial design | 3a | Description of trial design (such as parallel, factorial) including allocation ratio | 4 |
|  | 3b | Important changes to methods after trial commencement (such as eligibility criteria), with reasons | Not applicable |
| Participants | 4a | Eligibility criteria for participants | 4 |
|  | 4b | Settings and locations where the data were collected | Not applicable |
| Interventions | 5 | The interventions for each group with sufficient details to allow replication, including how and when they were actually administered | 5 |
| Outcomes | 6a | Completely defined pre-specified primary and secondary outcome measures, including how and when they were assessed | 5-6 |
|  | 6b | Any changes to trial outcomes after the trial commenced, with reasons | Not applicable |
| Sample size | 7a | How sample size was determined | Not applicable |
|  | 7b | When applicable, explanation of any interim analyses and stopping guidelines | Not applicable |
| Randomisation: |  |  |  |
| Sequence generation | 8a | Method used to generate the random allocation sequence | Not applicable |
|  | 8b | Type of randomisation; details of any restriction (such as blocking and block size) | Not applicable |
| Allocation concealment mechanism | 9 | Mechanism used to implement the random allocation sequence (such as sequentially numbered containers), describing any steps taken to conceal the sequence until interventions were assigned | 6 |
| Implementation | 10 | Who generated the random allocation sequence, who enrolled participants, and who assigned participants to interventions | Not applicable |
| Blinding | 11a | If done, who was blinded after assignment to interventions (for example, participants, care providers, those assessing outcomes) and how | 6 |
|  | 11b | If relevant, description of the similarity of interventions | Not applicable |
| Statistical methods | 12a | Statistical methods used to compare groups for primary and secondary outcomes | 6-7 |
|  | 12b | Methods for additional analyses, such as subgroup analyses and adjusted analyses | 7 |
| **Results** | | | |
| Participant flow (a diagram is strongly recommended) | 13a | For each group, the numbers of participants who were randomly assigned, received intended treatment, and were analysed for the primary outcome | 8 |
|  | 13b | For each group, losses and exclusions after randomisation, together with reasons | 8 |
| Recruitment | 14a | Dates defining the periods of recruitment and follow-up | 8 |
|  | 14b | Why the trial ended or was stopped | Not applicable |
| Baseline data | 15 | A table showing baseline demographic and clinical characteristics for each group | Supplemental materials |
| Numbers analysed | 16 | For each group, number of participants (denominator) included in each analysis and whether the analysis was by original assigned groups | 8 |
| Outcomes and estimation | 17a | For each primary and secondary outcome, results for each group, and the estimated effect size and its precision (such as 95% confidence interval) | 8-9 |
|  | 17b | For binary outcomes, presentation of both absolute and relative effect sizes is recommended | 8-9 |
| Ancillary analyses | 18 | Results of any other analyses performed, including subgroup analyses and adjusted analyses, distinguishing pre-specified from exploratory | 8-9 |
| Harms | 19 | All important harms or unintended effects in each group (for specific guidance see CONSORT for harms) | Not applicable |
| **Discussion** | | | |
| Limitations | 20 | Trial limitations, addressing sources of potential bias, imprecision, and, if relevant, multiplicity of analyses | 11-12 |
| Generalisability | 21 | Generalisability (external validity, applicability) of the trial findings | 12 |
| Interpretation | 22 | Interpretation consistent with results, balancing benefits and harms, and considering other relevant evidence | 9-11 |
| **Other information** | | |  |
| Registration | 23 | Registration number and name of trial registry | 4 |
| Protocol | 24 | Where the full trial protocol can be accessed, if available | Not applicable |
| Funding | 25 | Sources of funding and other support (such as supply of drugs), role of funders | 13 |

#

# **Expanded Methods**

**(1) Adjusted Analyses**

To avoid non-convergence when all adjusted covariates were introduced into the adjusted analyses simultaneously, we calculated a propensity score with treatment as the dependent variable and all adjusted covariates listed above as independent variables through a logistic regression model, and then included the calculated propensity score (continuous variable) as a covariate in the model for adjusted analysis.

**(2) Missing Data Imputation**

Missing data of covariates included in the adjusted analyses were imputed through simple imputation. Briefly, missing values for continuous variables were imputed from random values assuming a normal distribution with mean and standard deviation calculated from the available sample, and missing values for count variables were imputed from random values from a Poisson distribution with mean λ estimated from the available sample.

**(3) Sensitivity Analysis**

Propensity score matching was performed to generate a new cohort with balanced sample size in each category. Baseline characteristics including age, gender, time from symptom onset to treatment, premorbid function, history of stroke or transient ischemic attack, baseline NIHSS score, and potential confounders with P value <0.1 between categories were matched with the ratio 1:1, the caliper of 0.05, and a nearest-neighbor matching strategy. Then, we estimated the treatment effects on primary outcome in each matched category and the interaction between categories.

# **Table S1 Baseline Characteristics between Different Analysis Sets in RICAMIS.**

|  | **Per-protocol analysis set**  **(N=1707)** | **Full analysis set (N=1776)** | ***P* Value** |
| --- | --- | --- | --- |
| Age, y | 65 (58-73) | 65 (58-73) | 0.96 |
| Sex (F) | 579 (33.9) | 606 (34.1) | 0.90 |
| Current smoker | 475/1648 (28.8) | 505/1717 (29.4) | 0.71 |
| Current drinker ^a^ | 222/1666 (13.3) | 240/1735 (13.8) | 0.67 |
| Comorbidities ^b^ | | | |
| Hypertension | 1038/1684 (61.6) | 1083/1753 (61.8) | 0.93 |
| Diabetes | 406 (23.9) | 24.4/1770 (24.4) | 0.74 |
| Previous stroke ^c^ | 550/1696 (32.4) | 569/1765 (32.2) | 0.90 |
| Previous TIA | 18/1703 (1.1) | 22/1772 (1.2) | 0.61 |
| Blood pressure at randomization, mmHg | | | |
| Systolic | 150 (140-165) | 150 (140-165) | 0.92 |
| Diastolic | 90 (80-98) | 90 (80-98) | 0.93 |
| FBG at randomization, mmol/L | 6.26 (5.40-8.20) | 6.28 (5.40-8.28) | 0.80 |
| NIHSS score at randomization ^d^ | 7 (6-9) | 7 (6-9) | 0.72 |
| Estimated premorbid function (mRS score) ^e^ | | | |
| No symptoms (score, 0) | 1273 (74.6) | 1332 (75.0) | 0.77 |
| Symptoms without any disability (score, 1) | 434 (25.4) | 444 (25.0) |  |
| OTT, h | 25.5 (13.3-35.3) | 25.2 (13.0-35.0) | 0.70 |
| Duration of hospitalization, d ^g^ | 11 (10-12) | 11 (10-12) | 0.05 |
| Presumed stroke cause ^f^ | | | |
| Undetermined | 897/1705 (52.6) | 929/1773 (52.4) | 0.99 |
| Large artery atherosclerosis | 492/1705 (28.9) | 516/1773 (29.1) |  |
| Small artery occlusion | 275/1705 (16.1) | 284/1773 (16.0) |  |
| Other | 22/1705 (1.3) | 22/1773 (1.2) |  |
| Cardioembolic | 19/1705 (1.1) | 22/1773 (1.2) |  |

The data was shown with median (interquartile range) or number (percentage).

Abbreviation: FBG, fasting blood glucose; mRS, modified Rankin Scale; NIHSS indicates National Institute of Health Stroke Scale; OTT, time from onset of symptom to treatment; RIC, remote ischemic conditioning; TIA, transient ischemic attack.

^a^ Defined as consuming alcohol at least once a week within 1 year prior to the onset of the disease.

^b^ The comorbidities were based on the patient or family report.

^c^ Previous stroke included ischemic and hemorrhagic stroke. Previous ischemic stroke referred only to the patients with pre-stroke mRS ≤ 1.

^d^ Patients with NIHSS scores of 6 to 16 were eligible for this study; NIHSS scores range from 0 to 42, with higher scores indicating more severe neurological deficit.

^e^ Scores on the mRS of functional disability range from 0 (no symptoms) to 6 (death).

^f^ The presumed stroke cause was classified according to the Trial of ORG10172 in Acute Stroke Treatment (TOAST) using clinical findings, brain imaging, and laboratory test results. Other causes included nonatherosclerotic vasculopathies, hypercoagulable states, and hematologic disorder.

^g^ The duration of hospitalization were compared using rank-sum test due to the different distribution between analysis sets.

# **Table S2 Baseline Characteristics between Groups in the Current Analysis.**

|  | **RIC group**  **(N=808)** | **Control group**  **(N=899)** | ***P* Value** |
| --- | --- | --- | --- |
| Age, y | 65 (58-72) | 66 (58-73) | 0.75 |
| Sex (F) | 288 (35.6) | 291 (32.4) | 0.15 |
| Current smoker | 231/784 (29.5) | 244/864 (28.2) | 0.58 |
| Current drinker ^a^ | 119/793 (15.0) | 103/873 (11.8) | 0.05 |
| Comorbidities ^b^ | | | |
| Hypertension | 496/797 (62.2) | 542/887 (61.1) | 0.63 |
| Diabetes | 171/807 (23.7) | 215/894 (24.0) | 0.85 |
| Previous stroke ^c^ | 263/803 (32.8) | 287/893 (32.1) | 0.79 |
| Previous TIA | 8/806 (1.0) | 10/897 (1.1) | 0.81 |
| Systolic blood pressure, mmHg | | | |
| At admission | 150 (140-163) | 150 (140-165) | 0.31 |
| 7 days | 140 (130-150) | 140 (130-150) | 0.74 |
| 12 days | 138 (130-142) | 137 (130-142) | 0.61 |
| Diastolic blood pressure, mmHg | | | |
| At admission | 90 (80-98) | 88 (80-97) | 0.20 |
| 7 days | 80 (79-90) | 80 (78-90) | 0.43 |
| 12 days | 80 (78-87) | 80 (78-85) | 0.14 |
| Mean artery pressure, mmHg | | | |
| At admission | 110 (101-118) | 110 (102-119) | 0.22 |
| 7 days | 101 (97-107) | 101 (96-107) | 0.79 |
| 12 days | 100 (96-105) | 100 (95-104) | 0.24 |
| FBG at randomization, mmol/L | 6.20 (5.38-8.09) | 6.35 (5.40-8.30) | 0.19 |
| NIHSS score at randomization ^d^ | 7 (6-9) | 7 (6-9) | 0.83 |
| Estimated premorbid function (mRS score) ^e^ | | | |
| No symptoms (score, 0) | 602 (74.5) | 671 (74.6) | 0.95 |
| Symptoms without any disability (score, 1) | 206 (25.5) | 228 (25.4) |  |
| OTT, h | 25.8 (14.0-34.6) | 25.3 (12.7-35.5) | 0.40 |
| Duration of hospitalization, d | 11 (10-12) | 11 (10-12) | 0.80 |
| Presumed stroke cause ^f^ | | | |
| Undetermined | 461 (57.1) | 436/897 (48.6) | 0.002 |
| Large artery atherosclerosis | 212 (26.2) | 280/897 (31.2) |  |
| Small artery occlusion | 114 (14.1) | 161/897 (17.9) |  |
| Other | 14 (1.7) | 8/897 (0.9) |  |
| Cardioembolic | 7 (0.9) | 12/897 (1.3) |  |
| Previous use of antihypertension drug | 496/797 (62.2) | 542/887 (61.1) | 0.63 |
| Use of antihypertension drug in hospital | 332 (41.1) | 335 (37.3) | 0.11 |

The data was shown with median (interquartile range) or number (percentage).

Abbreviation: FBG, fasting blood glucose; mRS, modified Rankin Scale; NIHSS indicates National Institute of Health Stroke Scale; OTT, time from onset of symptom to treatment; RIC, remote ischemic conditioning; TIA, transient ischemic attack.

^a^ Defined as consuming alcohol at least once a week within 1 year prior to the onset of the disease.

^b^ The comorbidities were based on the patient or family report.

^c^ Previous stroke included ischemic and hemorrhagic stroke. Previous ischemic stroke referred only to the patients with pre-stroke mRS ≤ 1.

^d^ Patients with NIHSS scores of more than or equal to 6 were eligible for this study; NIHSS scores range from 0 to 42, with higher scores indicating more severe neurological deficit.

^e^ Scores on the mRS of functional disability range from 0 (no symptoms) to 6 (death).

^f^ The presumed stroke cause was classified according to the Trial of ORG10172 in Acute Stroke Treatment (TOAST) using clinical findings, brain imaging, and laboratory test results. Other causes included nonatherosclerotic vasculopathies, hypercoagulable states, and hematologic disorder.

# **Table S3 Baseline Characteristics between Treatment Groups in Different Max-Min Categories.**

|  | **High Max-Min (N=990)** | | | **Low Max-Min (N=717)** | | | ***P* Value ^g^** |
| --- | --- | --- | --- | --- | --- | --- | --- |
|  | **RIC group**  **(N=474)** | **Control group**  **(N=516)** | ***P* Value** | **RIC group**  **(N=334)** | **Control group**  **(N=383)** | ***P* Value** |  |
| Age, y | 66 (58-73) | 65 (59-73) | 0.501 | 65 (57-72) | 66 (59-74) | 0.300 | 0.886 |
| Sex (F) | 190 (40.1) | 174 (33.7) | 0.038 | 98 (29.3) | 117 (30.5) | 0.725 | 0.003 |
| Current smoker | 129/458 (28.2) | 153/494 (31.0) | 0.343 | 102/326 (31.3) | 91/370 (24.6) | 0.049 | 0.402 |
| Current drinker ^a^ | 64/464 (13.8) | 53/502 (10.6) | 0.124 | 55/329 (16.7) | 50/371 (13.5) | 0.231 | 0.087 |
| Comorbidities ^b^ | | | | | | | |
| Hypertension | 307/467 (65.7) | 325/509 (63.9) | 0.537 | 189/330 (57.3) | 217/378 (57.4) | 0.971 | 0.002 |
| Diabetes | 103/474 (21.7) | 123/512 (24.0) | 0.392 | 88/333 (26.4) | 92/382 (24.1) | 0.472 | 0.282 |
| Previous stroke ^c^ | 141/471 (29.9) | 163/514 (31.7) | 0.547 | 122/332 (36.7) | 124/379 (32.7) | 0.260 | 0.105 |
| Previous TIA | 5/473 (1.1) | 4/515 (0.8) | 0.898 | 3/333 (0.9) | 6/382 (1.6) | 0.642 | 0.488 |
| Blood pressure at randomization, mmHg | | | | | | | |
| Systolic | 160 (150-170) | 160 (150-171) | 0.064 | 140 (130-150) | 140 (132-150) | 0.799 | <0.001 |
| Diastolic | 90 (83-100) | 90 (85-100) | 0.383 | 85 (80-90) | 85 (80-91) | 0.200 | <0.001 |
| FBG at randomization, mmol/L | 6.18 (5.46-7.91) | 6.30 (5.49-8.13) | 0.448 | 6.26 (5.30-8.21) | 6.37 (5.35-8.51) | 0.536 | 0.950 |
| NIHSS score at randomization ^d^ | 7 (6-9) | 7 (6-9) | 0.771 | 7 (6-9) | 7 (6-9) | 0.264 | 0.189 |
| Estimated premorbid function (mRS score) ^e^ | | | | | | |  |
| No symptoms (score, 0) | 365 (77.0) | 387 (75.0) | 0.461 | 237 (71.0) | 284 (74.2) | 0.339 | 0.123 |
| Symptoms without any disability (score, 1) | 109 (23.0) | 129 (25.0) |  | 97 (29.0) | 99 (25.8) |  |  |
| OTT, h | 26.1 (14.6-35.7) | 25.3 (13.0-35.5) | 0.934 | 26.0 (16.1-34.9) | 25.4 (12.9-35.2) | 0.940 | 0.850 |
| Duration of hospitalization, d | 11 (10-12) | 11 (10-12) | 0.886 | 11 (10-12) | 11 (10-12) | 0.884 | <0.001 |
| Presumed stroke cause ^f^ | | | | | | | |
| Undetermined | 274 (57.8) | 251 (48.8) | 0.030 | 187 (56.0) | 185 (48.3 | 0.215 | 0.560 |
| Large artery atherosclerosis | 119 (25.1) | 156 (30.4) |  | 93 (27.8) | 124 (32.4) |  |  |
| Small artery occlusion | 67 (14.1) | 94 (18.3) |  | 47 (14.1) | 67 (17.5) |  |  |
| Other | 10 (2.1) | 6 (1.2) |  | 4 (1.2) | 2 (0.5) |  |  |
| Cardioembolic | 4 (0.8) | 7 (1.4) |  | 3 (0.9) | 5 (1.3) |  |  |
| Previous use of antihypertension drug | 307/467 (65.7) | 325/509 (63.9) | 0.537 | 189/330 (57.3) | 217/378 (57.4) | 0.971 | 0.06 |
| Use of antihypertension drug in hospital | 210 (44.3) | 208 (40.3) | 0.204 | 122 (36.5) | 127 (33.2) | 0.345 | 0.07 |

The data was shown with median (interquartile range) or number (percentage).

Abbreviation: FBG, fasting blood glucose; mRS, modified Rankin Scale; NIHSS indicates National Institute of Health Stroke Scale; OTT, time from onset of symptom to treatment; RIC, remote ischemic conditioning; TIA, transient ischemic attack.

^a^ Defined as consuming alcohol at least once a week within 1 year prior to the onset of the disease.

^b^ The comorbidities were based on the patient or family report.

^c^ Previous stroke included ischemic and hemorrhagic stroke. Previous stroke referred only to the patients with pre-stroke mRS ≤ 1.

^d^ Patients with NIHSS scores of more than or equal to 6 were eligible for this study; NIHSS scores range from 0 to 42, with higher scores indicating more severe neurological deficit.

^e^ Scores on the modified Rankin Scale (mRS) of functional disability range from 0 (no symptoms) to 6 (death).

^f^ The presumed stroke cause was classified according to TOAST using clinical findings, brain imaging, and laboratory test results. Other causes included nonatherosclerotic vasculopathies, hypercoagulable states, and hematologic disorder.

^g^ Compared between High and Low Max-Min Categories.

# **Table S4 Baseline Characteristics between Treatment Groups in Different Standard Deviation Categories.**

|  | **High Standard Deviation (N=402)** | | | **Low Standard Deviation (N=1305)** | | | ***P* Value ^g^** |
| --- | --- | --- | --- | --- | --- | --- | --- |
|  | **RIC group**  **(N=188)** | **Control group**  **(N=214)** | ***P* Value** | **RIC group**  **(N=620)** | **Control group**  **(N=685)** | ***P* Value** |  |
| Age, y | 67 (60-74) | 66 (59-74) | 0.259 | 65 (57-72) | 66 (59-73) | 0.385 | 0.088 |
| Sex (F) | 92 (48.9) | 84 (39.3) | 0.051 | 196 (31.6) | 207 (30.2) | 0.586 | <0.001 |
| Current smoker | 50/180 (27.8) | 63/204 (30.9) | 0.505 | 181/604 (30.0) | 181/660 (27.4) | 0.318 | 0.765 |
| Current drinker ^a^ | 23/180 (12.8) | 20/208 (9.6) | 0.322 | 96/613 (15.7) | 83/665 (12.5) | 0.102 | 0.138 |
| Comorbidities ^b^ | | | | | | | |
| Hypertension | 128/186 (68.8) | 130/212 (61.3) | 0.118 | 368/611 (60.2) | 412/675 (61.0) | 0.767 | 0.135 |
| Diabetes | 44/188 (23.4) | 48/213 (22.5) | 0.836 | 147/619 (23.7) | 167/681 (24.5) | 0.744 | 0.619 |
| Previous stroke ^c^ | 57/186 (30.6) | 62/214 (29.0) | 0.715 | 206/617 (33.4) | 225/679 (33.1) | 0.924 | 0.190 |
| Previous TIA | 2/188 (1.1) | 2/213 (0.9) | 0.900 | 6/618 (1.0) | 8/684 (1.2) | 0.728 | 0.894 |
| Blood pressure at randomization, mmHg | | | | | | | |
| Systolic | 170 (160-179) | 170 (160-180) | 0.497 | 148 (139-158) | 147 (138-160) | 0.749 | <0.001 |
| Diastolic | 96 (88-100) | 97 (89-100) | 0.473 | 89 (80-95) | 90 (80-95) | 0.398 | <0.001 |
| FBG at randomization, mmol/L | 6.08 (5.39-8.04) | 6.40 (5.39-8.19) | 0.560 | 6.21 (5.38-8.10) | 6.30 (5.42-8.33) | 0.441 | 0.970 |
| NIHSS score at randomization ^d^ | 7 (6-9) | 7 (6-9) | 0.873 | 7 (6-9) | 7 (6-9) | 0.504 | 0.616 |
| Estimated premorbid function (mRS score) ^e^ | | | | | | | |
| No symptoms (score, 0) | 145 (77.1) | 165 (77.1) | 0.995 | 457 (73.7) | 506 (73.9) | 0.948 | 0.181 |
| Symptoms without any disability (score, 1) | 43 (22.9) | 49 (22.9) |  | 163 (26.3) | 179 (26.1) |  |  |
| OTT, h | 24.0 (14.2-34.8) | 24.4 (11.9-35.7) | 0.912 | 26.2 (16.5-35.5) | 26.0 (13.5-35.3) | 0.953 | 0.058 |
| Duration of hospitalization, d | 11 (10-11) | 11 (10-12) | 0.269 | 11 (10-12) | 11 (10-12) | 0.490 | <0.001 |
| Presumed stroke cause ^f^ | | | | | | | |
| Undetermined | 117 (62.2) | 88 (41.3) | <0.001 | 344 (55.5) | 348 (50.9) | 0.144 | 0.889 |
| Large artery atherosclerosis | 42 (22.3) | 77 (36.2) |  | 170 (27.4) | 203 (29.7) |  |  |
| Small artery occlusion | 27 (14.4) | 42 (19.7) |  | 87 (14.0) | 119 (17.4) |  |  |
| Other | 2 (1.1) | 2 (0.9) |  | 12 (1.9) | 6 (0.9) |  |  |
| Cardioembolic | 0 (0) | 4 (1.9) |  | 7 (1.1) | 8 (1.2) |  |  |
| Previous use of antihypertension drug | 128/186 (68.8) | 130/212 (61.3) | 0.118 | 368/611 (60.2) | 412/675 (61.0) | 0.767 | 0.135 |
| Use of antihypertension drug in hospital | 100 (53.2) | 97 (45.3) | 0.116 | 232 (37.4) | 238 (34.7) | 0.315 | 0.07 |

The data was shown with median (interquartile range) or number (percentage).

Abbreviation: FBG, fasting blood glucose; mRS, modified Rankin Scale; NIHSS indicates National Institute of Health Stroke Scale; OTT, time from onset of symptom to treatment; RIC, remote ischemic conditioning; TIA, transient ischemic attack.

^a^ Defined as consuming alcohol at least once a week within 1 year prior to the onset of the disease.

^b^ The comorbidities were based on the patient or family report.

^c^ Previous stroke included ischemic and hemorrhagic stroke. Previous stroke referred only to the patients with pre-stroke mRS ≤ 1.

^d^ Patients with NIHSS scores of more than or equal to 6 were eligible for this study; NIHSS scores range from 0 to 42, with higher scores indicating more severe neurological deficit.

^e^ Scores on the modified Rankin Scale (mRS) of functional disability range from 0 (no symptoms) to 6 (death).

^f^ The presumed stroke cause was classified according to TOAST using clinical findings, brain imaging, and laboratory test results. Other causes included nonatherosclerotic vasculopathies, hypercoagulable states, and hematologic disorder.

^g^ Compared between High and Low Standard Deviation Categories.

# **Table S5 Baseline Characteristics between Treatment Groups in Different Coefficient of Variation Categories.**

|  | **High Coefficient of Variation (N=401)** | | | **Low Coefficient of Variation (N=1306)** | | | ***P* Value ^g^** |
| --- | --- | --- | --- | --- | --- | --- | --- |
|  | **RIC group**  **(N=189)** | **Control group**  **(N=212)** | ***P* Value** | **RIC group**  **(N=619)** | **Control group**  **(N=687)** | ***P* Value** |  |
| Age, y | 66 (60-74) | 66 (59-74) | 0.244 | 65 (57-72) | 66 (59-73) | 0.380 | 0.254 |
| Sex (F) | 89 (47.1) | 84 (39.6) | 0.132 | 199 (32.1) | 207 (30.1) | 0.432 | <0.001 |
| Current smoker | 50/180 (27.8) | 60/202 (29.7) | 0.678 | 181/604 (30.0) | 184/662 (27.8) | 0.394 | 0.989 |
| Current drinker ^a^ | 24/181 (13.3) | 19/206 (9.2) | 0.207 | 95/612 (15.5) | 84/667 (12.6) | 0.131 | 0.144 |
| Comorbidities ^b^ | | | | | | | |
| Hypertension | 129/188 (68.6) | 126/209 (60.3) | 0.084 | 367/609 (60.3) | 416/678 (61.4) | 0.688 | 0.224 |
| Diabetes | 43/189 (22.8) | 47/211 (22.3) | 0.909 | 148/618 (23.9) | 168/683 (24.6) | 0.785 | 0.463 |
| Previous stroke ^c^ | 59/187 (31.6) | 57/212 (26.9) | 0.306 | 204/616 (33.1) | 230/681 (33.8) | 0.802 | 0.101 |
| Previous TIA | 2/189 (1.1) | 3/211 (1.4) | 1.000 | 6/617 (1.0) | 7/686 (1.0) | 0.931 | 0.666 |
| Blood pressure at randomization, mmHg | | | | | | | |
| Systolic | 169 (158-178) | 170 (160-179) | 0.496 | 148 (139-159) | 149 (138-160) | 0.541 | <0.001 |
| Diastolic | 95 (85-100) | 96 (87-100) | 0.287 | 90 (80-95) | 90 (80-95) | 0.450 | <0.001 |
| FBG at randomization, mmol/L | 6.01 (5.36-7.92) | 6.40 (5.38-8.20) | 0.382 | 6.25 (5.38-8.11) | 6.30 (5.42-8.33) | 0.546 | 0.758 |
| NIHSS score at randomization ^d^ | 7 (6-9) | 7 (6-9) | 0.908 | 7 (6-9) | 7 (6-9) | 0.517 | 0.446 |
| Estimated premorbid function (mRS score) ^e^ | | | | | | | |
| No symptoms (score, 0) | 146 (77.2) | 167 (78.8) | 0.713 | 456 (73.7) | 504 (73.4) | 0.901 | 0.067 |
| Symptoms without any disability (score, 1) | 43 (22.8) | 45 (21.2) |  | 163 (26.3) | 183 (26.6) |  |  |
| OTT, h | 24.0 (13.6-33.8) | 25.0 (13.0-35.9) | 0.605 | 26.2 (16.8-36.0) | 26.0 (12.9-35.3) | 0.678 | 0.085 |
| Duration of hospitalization, d | 10 (10-11) | 10 (10-12) | 0.300 | 11 (10-12) | 11 (10-12) | 0.512 | <0.001 |
| Presumed stroke cause ^f^ | | | | | | | |
| Undetermined | 119 (63.0) | 86 (40.8) | <0.001 | 342 (55.3) | 350 (51.0) | 0.199 | 0.899 |
| Large artery atherosclerosis | 43 (22.8) | 75 (35.5) |  | 169 (27.3) | 205 (29.9) |  |  |
| Small artery occlusion | 25 (13.2) | 44 (20.9) |  | 89 (14.4) | 117 (17.1) |  |  |
| Other | 2 (1.1) | 2 (0.9) |  | 12 (1.9) | 6 (0.9) |  |  |
| Cardioembolic | 0 (0) | 4 (1.9) |  | 7 (1.1) | 8 (1.2) |  |  |
| Previous use of antihypertension drug | 129/188 (68.6) | 126/209 (60.3) | 0.084 | 367/609 (60.3) | 416/678 (61.4) | 0.688 | 0.224 |
| Use of antihypertension drug in hospital | 94 (49.7) | 91 (42.9) | 0.172 | 238 (38.4) | 244 (35.5) | 0.273 | 0.08 |

The data was shown with median (interquartile range) or number (percentage).

Abbreviation: FBG, fasting blood glucose; mRS, modified Rankin Scale; NIHSS indicates National Institute of Health Stroke Scale; OTT, time from onset of symptom to treatment; RIC, remote ischemic conditioning; TIA, transient ischemic attack.

^a^ Defined as consuming alcohol at least once a week within 1 year prior to the onset of the disease.

^b^ The comorbidities were based on the patient or family report.

^c^ Previous stroke included ischemic and hemorrhagic stroke. Previous stroke referred only to the patients with pre-stroke mRS ≤ 1.

^d^ Patients with NIHSS scores of more than or equal to 6 were eligible for this study; NIHSS scores range from 0 to 42, with higher scores indicating more severe neurological deficit.

^e^ Scores on the modified Rankin Scale (mRS) of functional disability range from 0 (no symptoms) to 6 (death).

^f^ The presumed stroke cause was classified according to TOAST using clinical findings, brain imaging, and laboratory test results. Other causes included nonatherosclerotic vasculopathies, hypercoagulable states, and hematologic disorder.

^g^ Compared between High and Low Coefficient of Variation Categories.

# **Table S6 Baseline Characteristics between Treatment Groups in Different Max-Min Categories after PSM.**

|  | **High Max-Min (N=828)** | | | **Low Max-Min (N=646)** | | | ***P* Value ^g^** |
| --- | --- | --- | --- | --- | --- | --- | --- |
|  | **RIC group**  **(N=414)** | **Control group**  **(N=414)** | ***P* Value** | **RIC group**  **(N=323)** | **Control group**  **(N=323)** | ***P* Value** |  |
| Age, y | 66 (58-72) | 65 (59-72) | 0.785 | 65 (58-73) | 66 (58-73) | 0.978 | 0.594 |
| Sex (F) | 158 (38.2) | 155 (37.4) | 0.830 | 98 (30.3) | 90 (27.9) | 0.488 | <0.001 |
| Current smoker | 122/400 (30.5) | 114/399 (28.6) | 0.550 | 91/315 (28.9) | 91/315 (28.9) | 1.000 | 0.789 |
| Current drinker ^a^ | 63/406 (15.5) | 43/404 (10.6) | 0.040 | 49/318 (15.4) | 49/313 (15.7) | 0.932 | 0.187 |
| Comorbidities ^b^ | | | | | | | |
| Hypertension | 270/409 (66.0) | 264/409 (64.5) | 0.659 | 185/319 (58.0) | 185/321 (57.6) | 0.926 | 0.004 |
| Diabetes | 88/414 (21.3) | 101/410 (24.6) | 0.249 | 86/322 (26.7) | 76/322 (23.6) | 0.364 | 0.323 |
| Previous stroke ^c^ | 125/412 (30.3) | 126/412 (30.6) | 0.940 | 120/321 (37.4) | 99/320 (30.9) | 0.085 | 0.132 |
| Previous TIA | 5/413 (1.2) | 3/413 (0.7) | 0.722 | 3/322 (0.9) | 6/322 (1.9) | 0.502 | 0.445 |
| Blood pressure at randomization, mmHg | | | | | | | |
| Systolic | 160 (150-170) | 160 (150-170) | 0.959 | 140 (130-150) | 140 (132-150) | 0.515 | <0.001 |
| Diastolic | 90 (84-100) | 90 (85-100) | 0.861 | 85 (80-90) | 87 (80-92) | 0.023 | <0.001 |
| FBG at randomization, mmol/L | 6.19 (5.50-7.75) | 6.39 (5.49-8.09) | 0.580 | 6.39 (5.30-8.30) | 6.40 (5.35-8.51) | 0.555 | 0.709 |
| NIHSS score at randomization ^d^ | 7 (6-9) | 7 (6-9) | 0.884 | 7 (6-9) | 7 (6-9) | 0.226 | 0.217 |
| Estimated premorbid function (mRS score) ^e^ | | | | | | |  |
| No symptoms (score, 0) | 313 (75.6) | 309 (74.6) | 0.748 | 227 (70.3) | 244 (75.5) | 0.132 | 0.336 |
| Symptoms without any disability (score, 1) | 101 (24.4) | 105 (25.4) |  | 96 (29.7) | 79 (24.5) |  |  |
| OTT, h | 26.0 (14.4-35.5) | 25.2 (12.4-34.8) | 0.917 | 26.0 (16.0-34.9) | 25.4 (12.7-35.3) | 0.908 | 0.516 |
| Duration of hospitalization, d | 11 (10-12) | 11 (10-12) | 0.556 | 11 (10-12) | 11 (10-12) | 0.582 | 0.003 |
| Presumed stroke cause ^f^ | | | | | | | |
| Undetermined | 223 (53.9) | 215 (52.2) | 0.314 | 179 (55.4) | 156 (48.3) | 0.262 | 0.618 |
| Large artery atherosclerosis | 112 (27.1) | 117 (28.4) |  | 93 (28.8) | 101 (31.3) |  |  |
| Small artery occlusion | 65 (15.7) | 74 (18.0) |  | 44 (13.6) | 59 (18.3) |  |  |
| Other | 10 (2.4) | 3 (0.7) |  | 4 (1.2) | 2 (0.6) |  |  |
| Cardioembolic | 4 (1.0) | 3 (0.7) |  | 3 (0.9) | 5 (1.5) |  |  |

The data was shown with median (interquartile range) or number (percentage).

Abbreviation: FBG, fasting blood glucose; mRS, modified Rankin Scale; NIHSS indicates National Institute of Health Stroke Scale; OTT, time from onset of symptom to treatment; RIC, remote ischemic conditioning; TIA, transient ischemic attack.

^a^ Defined as consuming alcohol at least once a week within 1 year prior to the onset of the disease.

^b^ The comorbidities were based on the patient or family report.

^c^ Previous stroke included ischemic and hemorrhagic stroke. Previous stroke referred only to the patients with pre-stroke mRS ≤ 1.

^d^ Patients with NIHSS scores of more than or equal to 6 were eligible for this study; NIHSS scores range from 0 to 42, with higher scores indicating more severe neurological deficit.

^e^ Scores on the modified Rankin Scale (mRS) of functional disability range from 0 (no symptoms) to 6 (death).

^f^ The presumed stroke cause was classified according to TOAST using clinical findings, brain imaging, and laboratory test results. Other causes included nonatherosclerotic vasculopathies, hypercoagulable states, and hematologic disorder.

^g^ Compared between High and Low Max-Min Categories.

# **Table S7 Baseline Characteristics between Treatment Groups in Different Standard Deviation Categories after PSM.**

|  | **High Standard Deviation (N=310)** | | | **Low Standard Deviation** **(N=1168)** | | | ***P* Value ^g^** |
| --- | --- | --- | --- | --- | --- | --- | --- |
|  | **RIC group**  **(N=155)** | **Control group**  **(N=155)** | ***P* Value** | **RIC group**  **(N=584)** | **Control group**  **(N=584)** | ***P* Value** |  |
| Age, y | 66 (60-73) | 67 (60-74) | 0.820 | 65 (57-72) | 65 (58-72) | 0.774 | 0.028 |
| Sex (F) | 69 (44.5) | 70 (45.2) | 0.909 | 178 (30.5) | 185 (31.7) | 0.658 | <0.001 |
| Current smoker | 44/148 (29.7) | 41/149 (27.5) | 0.673 | 174/569 (30.6) | 151/562 (26.9) | 0.168 | 0.969 |
| Current drinker ^a^ | 20/148 (13.5) | 11/150 (7.3) | 0.081 | 92/578 (15.9) | 69/568 (12.1) | 0.066 | 0.099 |
| Comorbidities ^b^ | | | | | | | |
| Hypertension | 102/153 (66.7) | 97/155 (62.6) | 0.453 | 342/575 (59.5) | 355/574 (61.8) | 0.411 | 0.206 |
| Diabetes | 37/155 (23.9) | 36/154 (23.4) | 0.919 | 142/583 (24.4) | 145/580 (25.0) | 0.799 | 0.702 |
| Previous stroke ^c^ | 42/153 (27.5) | 46/155 (29.7) | 0.665 | 193/581 (33.2) | 193/580 (33.3) | 0.983 | 0.119 |
| Previous TIA | 2/155 (1.3) | 2/154 (1.3) | 1.000 | 6/582 (1.0) | 8/584 (1.4) | 0.595 | 0.894 |
| Blood pressure at randomization, mmHg | | | | | | | |
| Systolic | 170 (160-180) | 170 (160-180) | 0.956 | 148 (139-158) | 149 (138-160) | 0.333 | <0.001 |
| Diastolic | 97 (89-100) | 97 (88-100) | 0.907 | 89 (80-95) | 90 (80-95) | 0.103 | <0.001 |
| FBG at randomization, mmol/L | 6.10 (5.44-8.05) | 6.40 (5.30-8.50) | 0.920 | 6.25 (5.40-8.12) | 6.34 (5.46-8.34) | 0.353 | 0.892 |
| NIHSS score at randomization ^d^ | 7 (6-9) | 7 (6-9) | 0.996 | 7 (6-9) | 7 (6-9) | 0.766 | 0.801 |
| Estimated premorbid function (mRS score) ^e^ | | | | | | | |
| No symptoms (score, 0) | 119 (76.8) | 121 (78.1) | 0.786 | 432 (74.0) | 429 (73.5) | 0.842 | 0.184 |
| Symptoms without any disability (score, 1) | 36 (23.2) | 34 (21.9) |  | 152 (26.0) | 155 (26.5) |  |  |
| OTT, h | 23.8 (13.6-33.7) | 24.5 (12.7-35.1) | 0.677 | 26.2 (16.9-35.5) | 26.0 (13.4-34.9) | 0.954 | 0.076 |
| Duration of hospitalization, d | 11 (10-11) | 10 (10-11) | 0.953 | 11 (10-12) | 11 (10-12) | 0.542 | 0.001 |
| Presumed stroke cause ^f^ | | | | | | | |
| Undetermined | 95 (61.3) | 71 (45.8) | 0.018 | 321 (55.0) | 292 (50.1) | 0.271 | 0.706 |
| Large artery atherosclerosis | 37 (23.9) | 56 (36.1) |  | 161 (27.6) | 177 (30.4) |  |  |
| Small artery occlusion | 21 (13.5) | 26 (16.8) |  | 84 (14.4) | 101 (17.3) |  |  |
| Other | 2 (1.3) | 0 (0) |  | 11 (1.9) | 6 (1.0) |  |  |
| Cardioembolic | 0 (0) | 2 (1.3) |  | 7 (1.2) | 7 (1.2) |  |  |

The data was shown with median (interquartile range) or number (percentage).

Abbreviation: FBG, fasting blood glucose; mRS, modified Rankin Scale; NIHSS indicates National Institute of Health Stroke Scale; OTT, time from onset of symptom to treatment; RIC, remote ischemic conditioning; TIA, transient ischemic attack.

^a^ Defined as consuming alcohol at least once a week within 1 year prior to the onset of the disease.

^b^ The comorbidities were based on the patient or family report.

^c^ Previous stroke included ischemic and hemorrhagic stroke. Previous stroke referred only to the patients with pre-stroke mRS ≤ 1.

^d^ Patients with NIHSS scores of more than or equal to 6 were eligible for this study; NIHSS scores range from 0 to 42, with higher scores indicating more severe neurological deficit.

^e^ Scores on the modified Rankin Scale (mRS) of functional disability range from 0 (no symptoms) to 6 (death).

^f^ The presumed stroke cause was classified according to TOAST using clinical findings, brain imaging, and laboratory test results. Other causes included nonatherosclerotic vasculopathies, hypercoagulable states, and hematologic disorder.

^g^ Compared between High and Low Standard Deviation Categories.

# **Table S8 Baseline Characteristics between Treatment Groups in Different Coefficient of Variation Categories after PSM.**

|  | **High Coefficient of Variation (N=304)** | | | **Low Coefficient of Variation (N=1140)** | | | ***P* Value ^g^** |
| --- | --- | --- | --- | --- | --- | --- | --- |
|  | **RIC group**  **(N=152)** | **Control group**  **(N=152)** | ***P* Value** | **RIC group**  **(N=570)** | **Control group**  **(N=570)** | ***P* Value** |  |
| Age, y | 66 (59-72) | 66 (60-74) | 0.798 | 65 (58-73) | 66 (59-72) | 0.766 | 0.165 |
| Sex (F) | 62 (40.8) | 61 (40.1) | 0.907 | 177 (31.1) | 175 (30.7) | 0.898 | 0.002 |
| Current smoker | 46/144 (31.9) | 41/144 (28.5) | 0.521 | 170/556 (30.6) | 155/547 (28.3) | 0.415 | 0.806 |
| Current drinker ^a^ | 21/147 (14.3) | 13/146 (8.9) | 0.150 | 89/563 (15.8) | 69/552 (12.5) | 0.113 | 0.255 |
| Comorbidities ^b^ | | | | | | | |
| Hypertension | 103/152 (67.8) | 94/152 (61.8) | 0.280 | 336/560 (60.0) | 342/564 (60.6) | 0.827 | 0.155 |
| Diabetes | 38/152 (25.0) | 36/152 (23.7) | 0.789 | 137/569 (24.1) | 131/567 (23.1) | 0.699 | 0.785 |
| Previous stroke ^c^ | 40/151 (26.5) | 44/152 (28.9) | 0.633 | 188/567 (33.2) | 188/565 (33.3) | 0.967 | 0.069 |
| Previous TIA | 2/152 (1.3) | 1/151 (0.7) | 1.000 | 5/569 (0.9) | 7/569 (1.2) | 0.562 | 1.000 |
| Blood pressure at randomization, mmHg | | | | | | | |
| Systolic | 170 (160-178) | 170 (160-179) | 0.972 | 148 (140-159) | 149 (138-160) | 0.720 | <0.001 |
| Diastolic | 95 (87-100) | 96 (88-100) | 0.624 | 90 (80-95) | 90 (80-95) | 0.491 | <0.001 |
| FBG at randomization, mmol/L | 6.0 (5.37-7.92) | 6.50 (5.40-8.66) | 0.157 | 6.26 (5.38-8.20) | 6.29 (5.40-8.29) | 0.857 | 0.884 |
| NIHSS score at randomization ^d^ | 7 (6-9) | 7 (6-9) | 0.422 | 7 (6-9) | 7 (6-9) | 0.888 | 0.592 |
| Estimated premorbid function (mRS score) ^e^ | | | | | | | |
| No symptoms (score, 0) | 117 (77.0) | 122 (80.3) | 0.484 | 415 (72.8) | 423 (74.2) | 0.591 | 0.069 |
| Symptoms without any disability (score, 1) | 35 (23.0) | 30 (19.7) |  | 155 (27.2) | 147 (25.8) |  |  |
| OTT, h | 24.7 (14.3-34.4) | 24.0 (12.1-34.1) | 0.550 | 26.0 (16.2-35.3) | 26.5 (13.6-35.3) | 0.808 | 0.033 |
| Duration of hospitalization, d | 11 (10-12) | 10 (10-11) | 0.796 | 11 (10-12) | 11 (10-12) | 0.480 | 0.001 |
| Presumed stroke cause ^f^ | | | | | | | |
| Undetermined | 92 (60.5) | 64 (42.1) | 0.009 | 316 (55.4) | 291 (51.1) | 0.530 | 0.957 |
| Large artery atherosclerosis | 36 (23.7) | 57 (37.5) |  | 156 (27.4) | 173 (30.4) |  |  |
| Small artery occlusion | 22 (14.5) | 27 (17.8) |  | 83 (14.6) | 93 (16.3) |  |  |
| Other | 2 (1.3) | 1 (0.7) |  | 8 (1.4) | 5 (0.9) |  |  |
| Cardioembolic | 0 (0) | 3 (2.0) |  | 7 (1.2) | 7 (1.2) |  |  |

The data was shown with median (interquartile range) or number (percentage).

Abbreviation: FBG, fasting blood glucose; mRS, modified Rankin Scale; NIHSS indicates National Institute of Health Stroke Scale; OTT, time from onset of symptom to treatment; RIC, remote ischemic conditioning; TIA, transient ischemic attack.

^a^ Defined as consuming alcohol at least once a week within 1 year prior to the onset of the disease.

^b^ The comorbidities were based on the patient or family report.

^c^ Previous stroke included ischemic and hemorrhagic stroke. Previous stroke referred only to the patients with pre-stroke mRS ≤ 1.

^d^ Patients with NIHSS scores of more than or equal to 6 were eligible for this study; NIHSS scores range from 0 to 42, with higher scores indicating more severe neurological deficit.

^e^ Scores on the modified Rankin Scale (mRS) of functional disability range from 0 (no symptoms) to 6 (death).

^f^ The presumed stroke cause was classified according to TOAST using clinical findings, brain imaging, and laboratory test results. Other causes included nonatherosclerotic vasculopathies, hypercoagulable states, and hematologic disorder.

^g^ Compared between High and Low Coefficient of Variation Categories.
